# Supplementary material for: AAV delivery of GBA1 suppresses α-synuclein accumulation in Parkinson’s disease models and restores functions in Gaucher’s disease models
Source: PLoS One. 2025 May 7;20(5):e0321145. doi: 10.1371/journal.pone.0321145 (PMC12057913; doi:10.1371/journal.pone.0321145)
Supplement: S1 Fig — (A) Illustration of sampling scheme. (B) Illustration of the injection site (×) and positions where the dissections blades are inserted for tissue isolation. (C) Illustration of cross section of (2). (D) Illustration of cross section of (3). (E) Pictures of the positions of blades on a brain slicer and sampling procedures. (PDF) [file pone.0321145.s001.pdf]

S1 Fig.

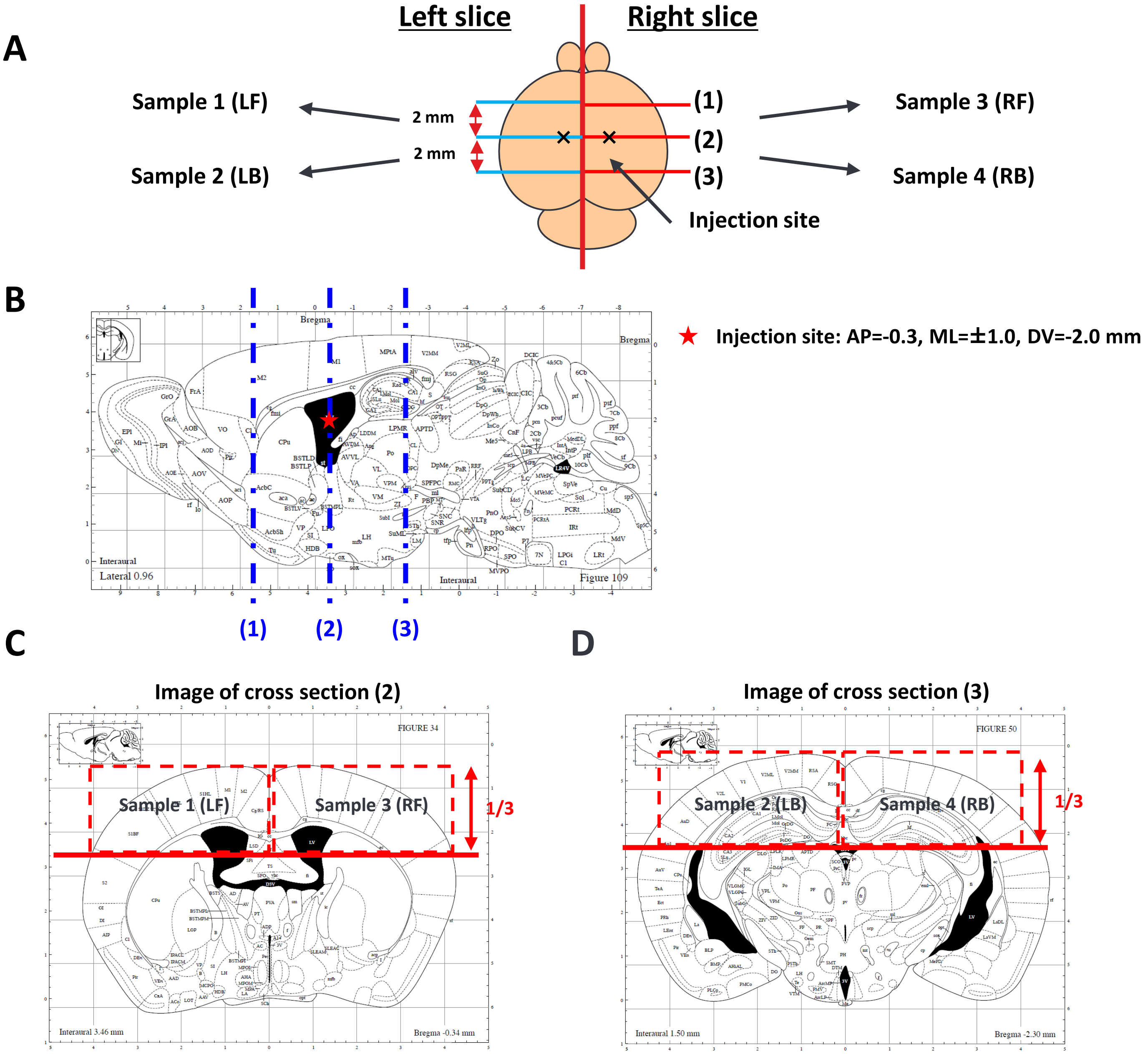

E

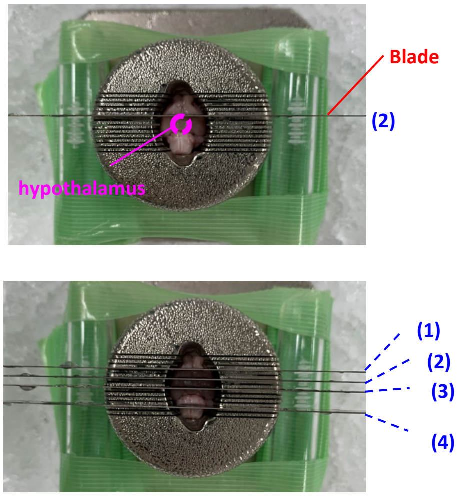

Step 1

Place the second blade (position (2)) so that it is just above the tip of the hypothalamus.
(This position is about 0.5 mm before the site of administration, so be careful not to insert it earlier than this.)

Step 2

Place the first (position (1)) and third (position (3)) blades 2 mm in front of and behind the second blade (skip one square in the brain matrix).

Placing another blade around the brainstem (position (4)) prevents the brain from shifting when cutting.

Step 3

When cutting, use a flat object (e.g. 15-ml tube) to push both ends of the blades evenly and all at once. Cutting one blade at a time can cause the brain to shift.
