# Supplementary figures and images for: AAV delivery of GBA1 suppresses α-synuclein accumulation in Parkinson’s disease models and restores functions in Gaucher’s disease models
Source: PLoS One. 2025 May 7;20(5):e0321145. doi: 10.1371/journal.pone.0321145 (PMC12057913; doi:10.1371/journal.pone.0321145)

S2 Fig.

A. Protein concentration of Fig. 1A

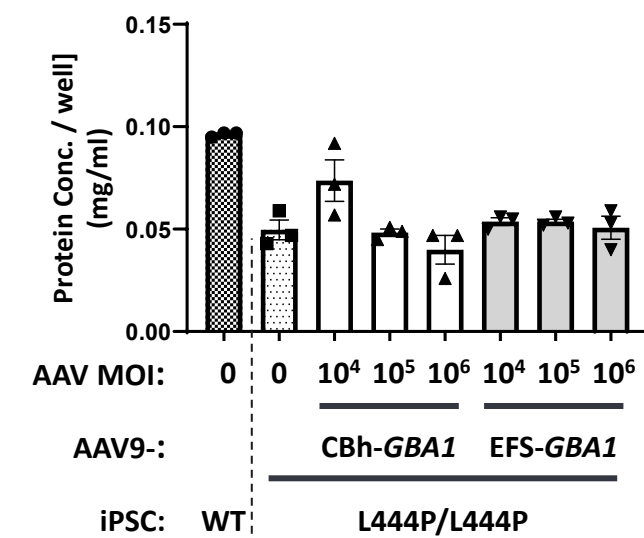

B. Protein concentration of Fig. 1C

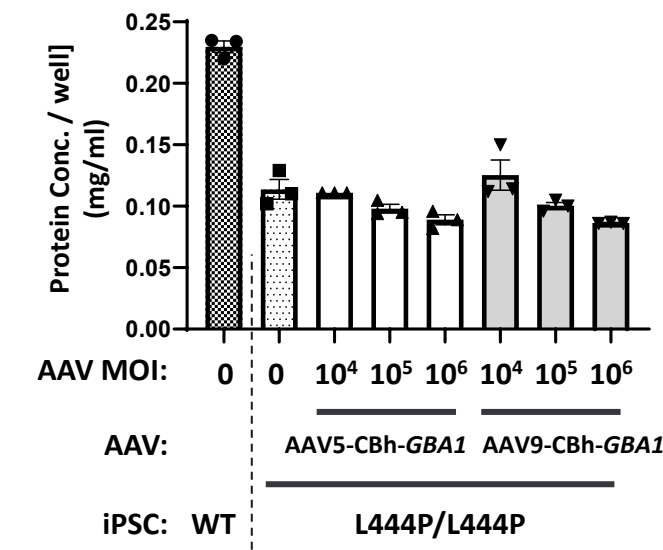

Supplement: S2 Fig — (A) Graph shows total protein concentration for human wild-type (WT), L444P/L444P GBA1 mutant iPSC-derived DA neurons, and L444P/L444P GBA1 mutant iPSC-derived DA neurons transduced with AAV vectors at three MOIs (1 × 10^4^, 1 × 10^5^, and 1 × 10^6^ VG) seven days after differentiation and analyzed two weeks after AAV transduction. The protein concentration in cell lysate in each well was quantified by using BCA protein assay Kit (mean ± SE (n = 3)). (B) As shown in Fig 2C, cells were transduced with AAV5-CBh-GBA1 or AAV9-CBh-GBA1. Then, the protein concentration in cell lysate in each well was quantified by using BCA protein assay Kit (mean ± SE (n = 3)). (PDF) [file pone.0321145.s002.pdf]

# S3 Fig.

## A. *hGBA1* mRNA expression

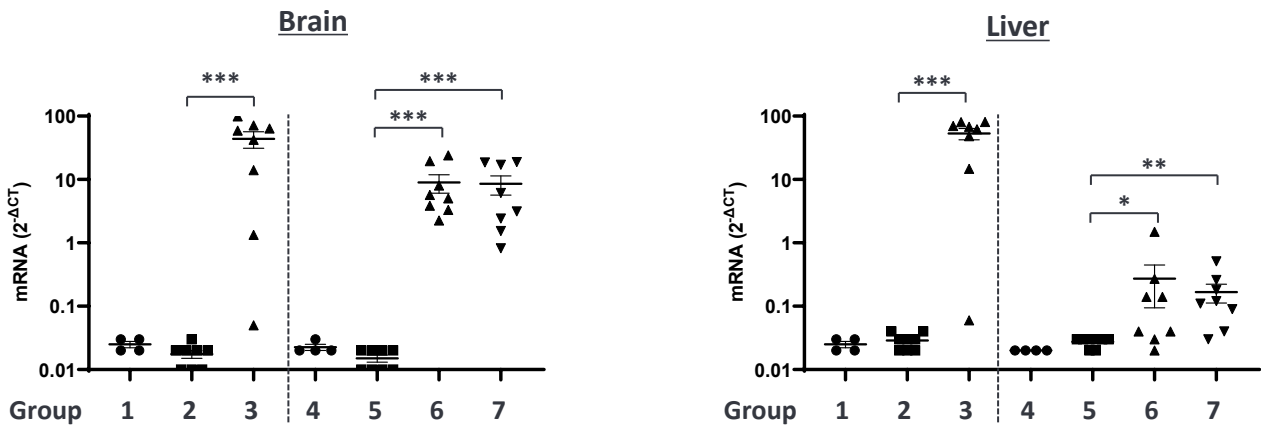

## B. Correlation between VG and mRNA

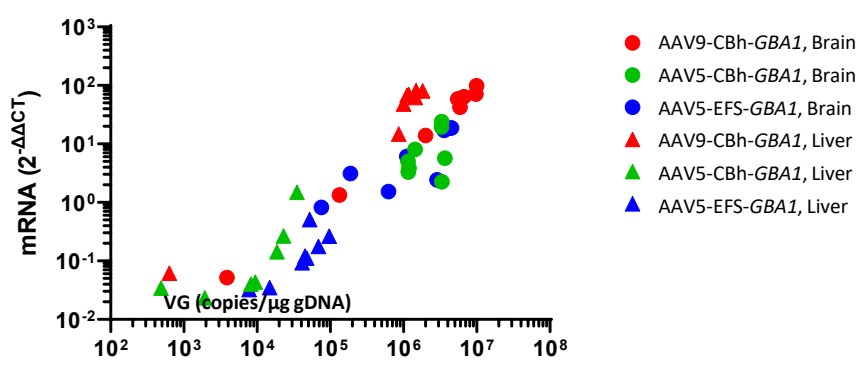

Supplement: S3 Fig — (A) Human GBA1 (hGBA1) mRNA in brain and liver was measured by using qPCR method. Each graph represents the mean ± S.E.M. (n = 4 or 8). The Y-axis shows logarithmic scale. Statistical analyses were performed by Dunnett analysis. *: < 0.05; **: < 0.01; ***: < 0.001, compared to Group 2 and Group 5 for batch 1 study and batch 2 study, respectively. (B) Correlation analysis between VG and mRNA. Both X-axis and Y-axis are logarithmic scale. (PDF) [file pone.0321145.s003.pdf]

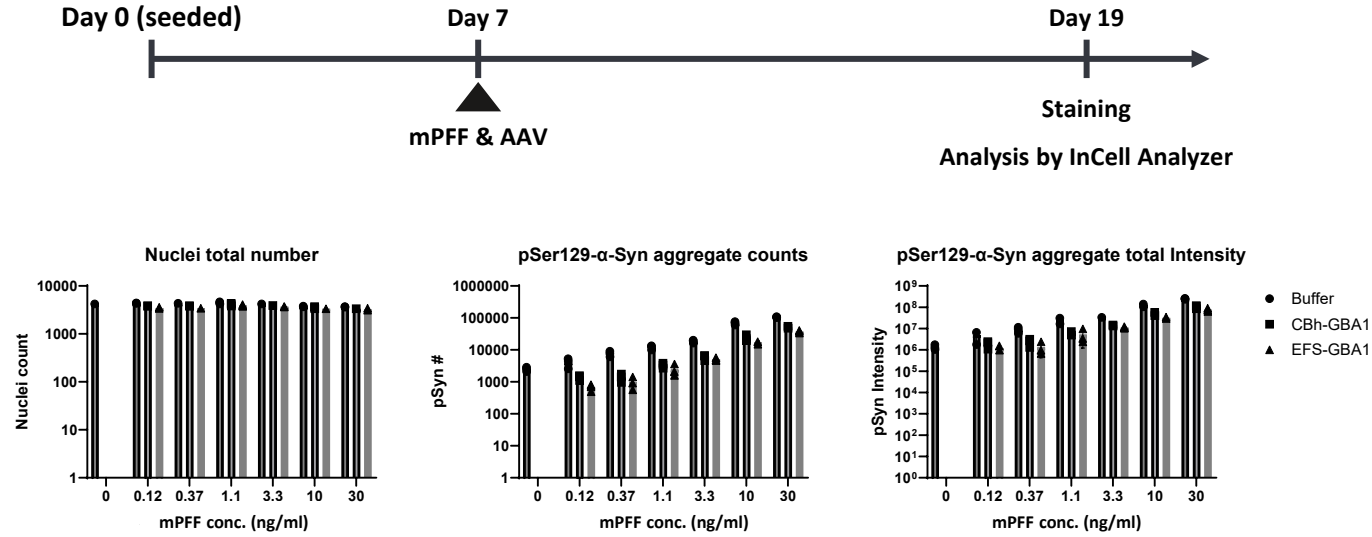

Supplement: S5 Fig — (A) The study layout is illustrated above. Mouse primary cortical neurons were seeded on poly-D-Lysin coated 96-well plate (45,000 cells/well) on Day 0. On Day 7, mouse pre-formed fibril (mPFF; 10-fold dilution series from 0.1 to 1,000 ng/ml) were added to the cells as noted on the x-axis. After 5- or 12-day culture, cells were stained with anti-phospho-α-synuclein antibody and analyzed by InCell Analyzer. Quantification of the nuclei counts, the pSer129 α-Syn aggregate counts, and the intensity of pSer129 α-Syn aggregates are shown. (B) The study layout is illustrated above. Mouse primary cortical neurons were treated with mPFF (3-fold dilution series from 0.12 to 30 ng/ml) and AAV9-CBh-GBA1 or AAV9-EFS-GBA1 (MOI at 1 × 10^5^ VG) were added to the cells as noted on the x-axis. After 12-day culture, cells were stained with anti-phospho-α-synuclein antibody and analyzed by InCell Analyzer. Quantification of the nuclei counts, the pSer129 α-Syn aggregate counts, and the intensity of pSer129 α-Syn aggregates are shown. Data are represented as mean ± S.E.M. (n = 3). Y-axis show logarithmic scale. (PDF) [file pone.0321145.s005.pdf]

S6 Fig.

A

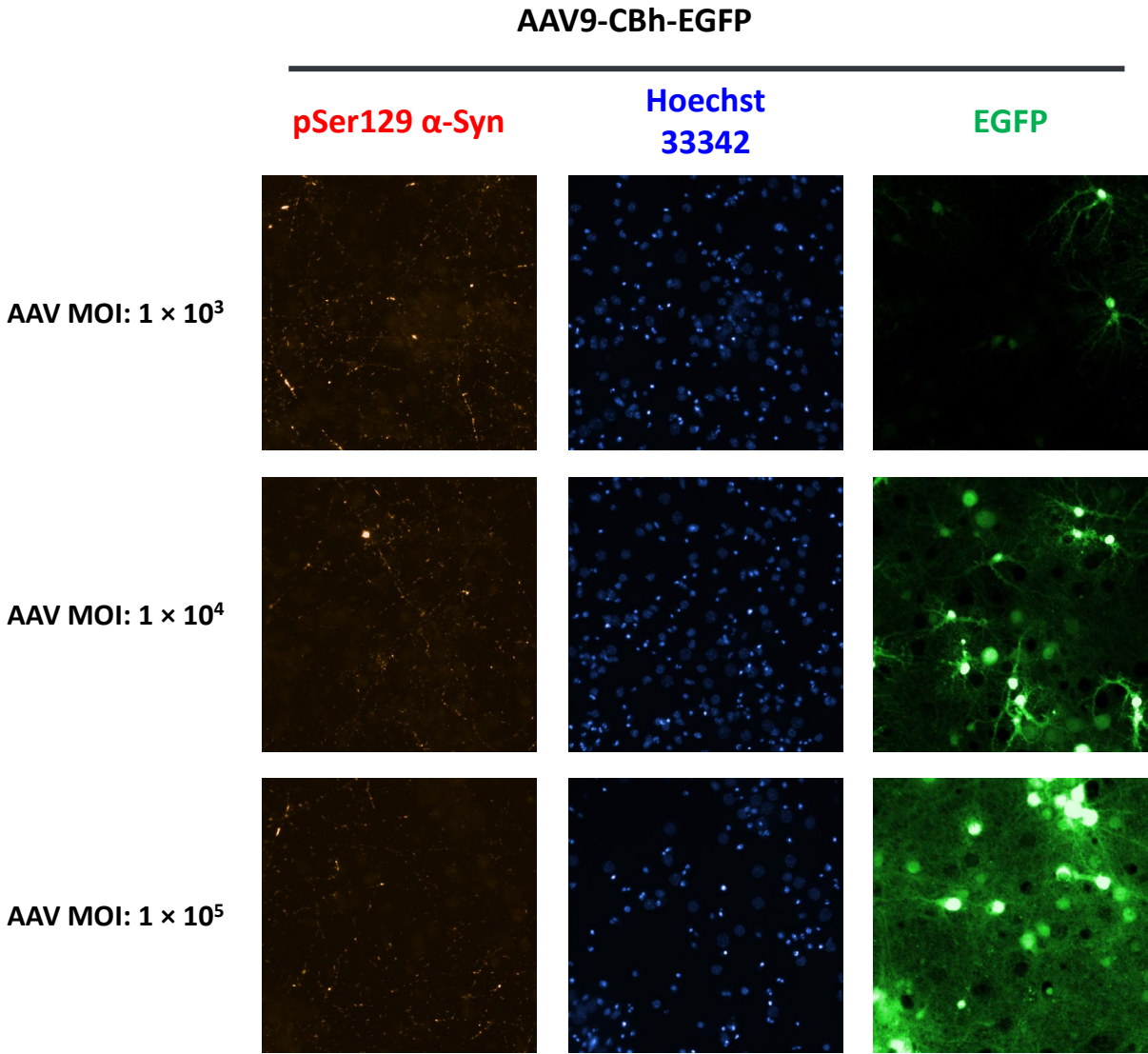

B

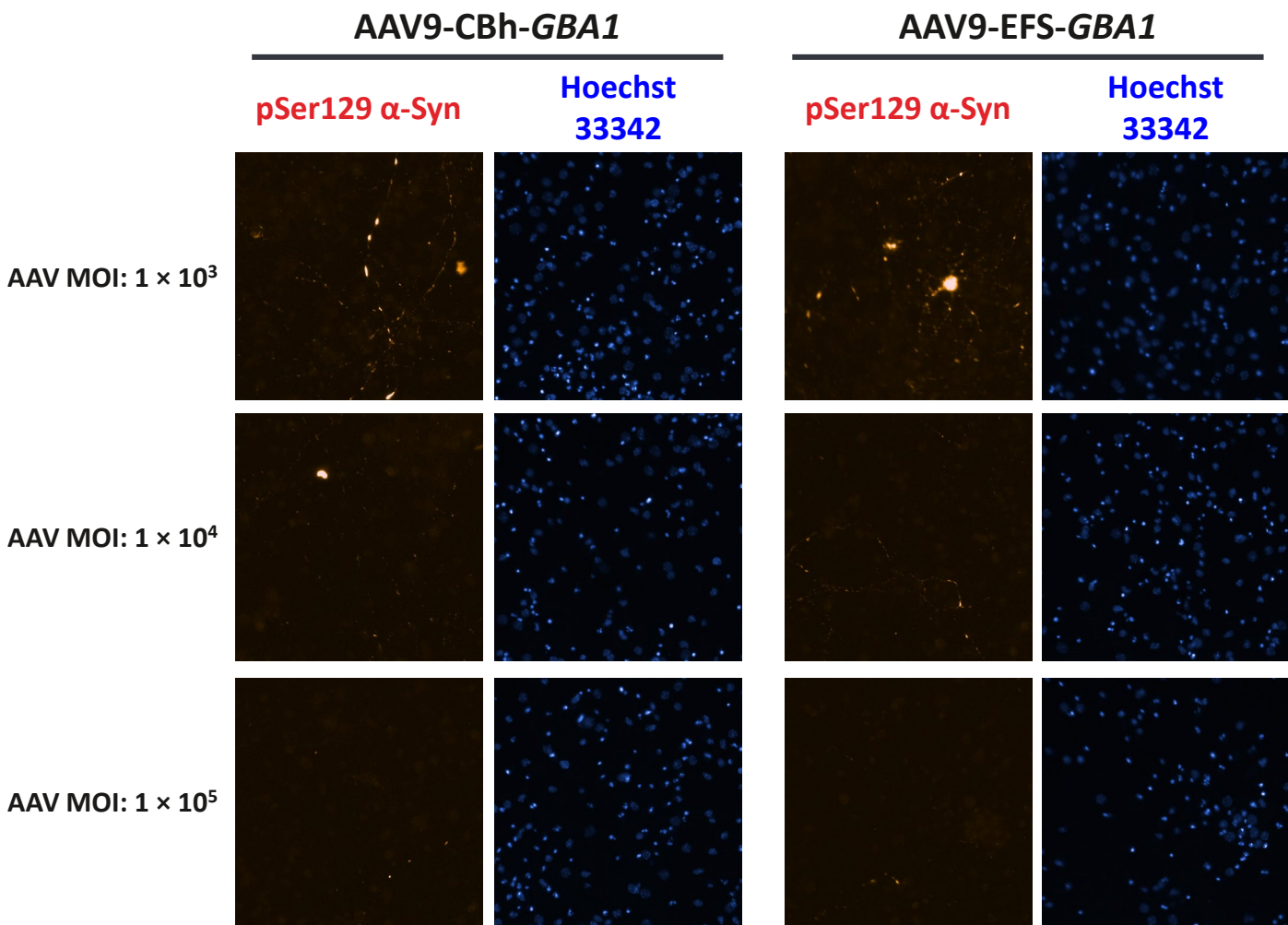

Supplement: S6 Fig — (A) Representative images of cells treated with AAV9-CBh-EGFP: pSer129 α-synuclein aggregates (red), nuclear (blue), and EGFP (green) are shown. (B) Representative images of cells treated with AAV9-CBh-GBA1 or AAV9-EFS-GBA1: pSer129 α-synuclein aggregates (red) and nuclear (blue) are shown. (PDF) [file pone.0321145.s006.pdf]
