# Supplementary material for: AAV delivery of GBA1 suppresses α-synuclein accumulation in Parkinson’s disease models and restores functions in Gaucher’s disease models
Source: PLoS One. 2025 May 7;20(5):e0321145. doi: 10.1371/journal.pone.0321145 (PMC12057913; doi:10.1371/journal.pone.0321145)

S4 Fig.

A. Body weight over time

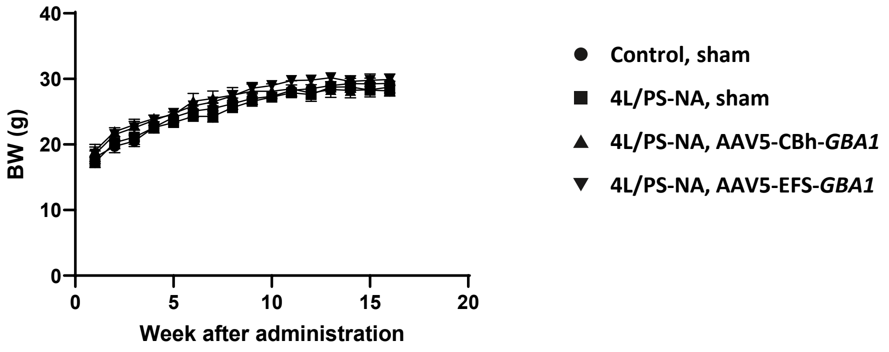

B. VG analysis

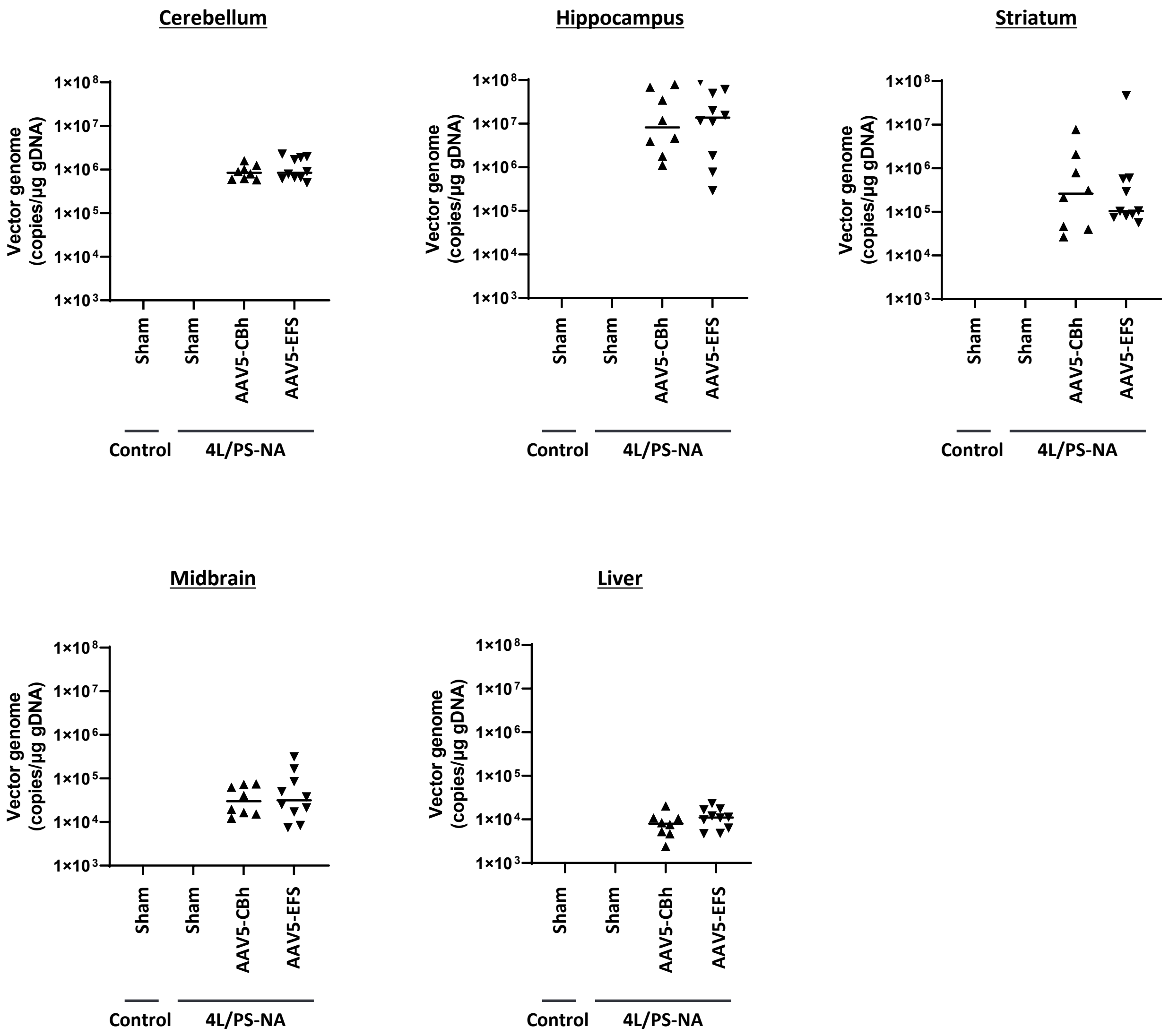

C. *hGBA1* mRNA expression

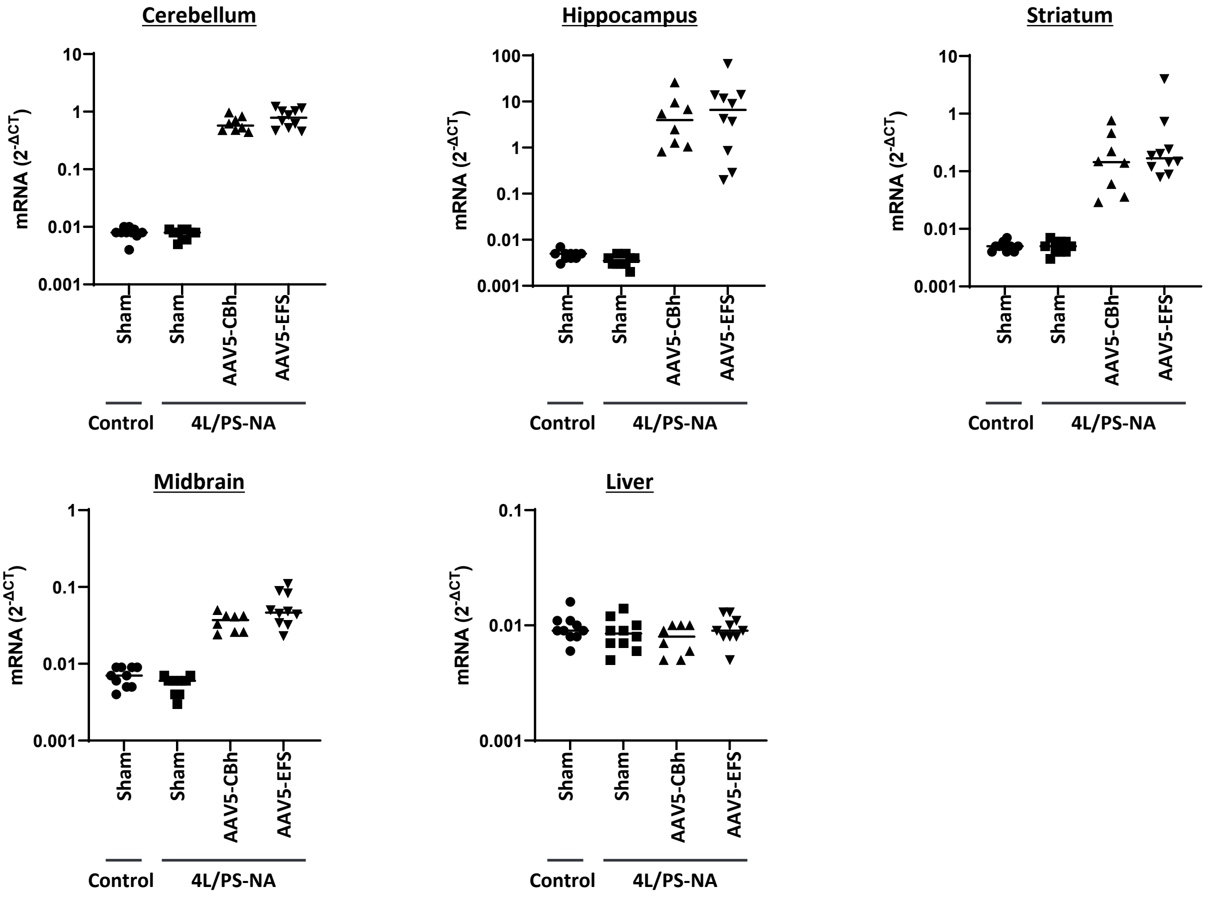

D. Correlation between VG and mRNA

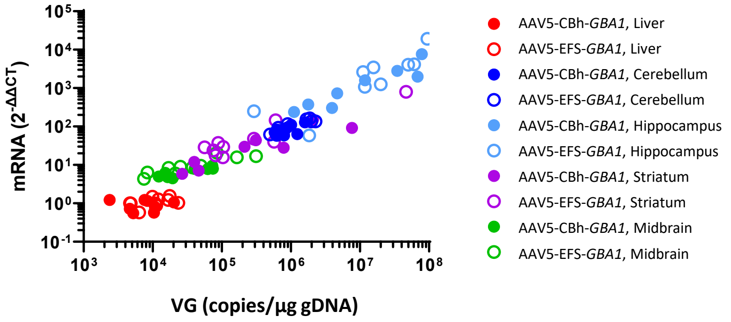

Supplement: S4 Fig — (A) Graph represents body weights per group over time.Animals that died prematurely were excluded from the analysis. (B) VG in brain and liver was measured by qPCR method. The Y-axis shows logarithmic scale. (C) HGBA1 mRNA in brain and liver was measured by using qPCR method. The Y-axis is logarithmic scale. Each graph represents the mean ± S.E.M. (n = 8–10). (D) Correlation analysis between VG and mRNA in each tissue. Both X-axis and Y-axis show logarithmic scale. (PDF) [file pone.0321145.s004.pdf]
