# Supplementary material for: AAV delivery of GBA1 suppresses α-synuclein accumulation in Parkinson’s disease models and restores functions in Gaucher’s disease models
Source: PLoS One. 2025 May 7;20(5):e0321145. doi: 10.1371/journal.pone.0321145 (PMC12057913; doi:10.1371/journal.pone.0321145)

S7 Fig.

A. Treatment Groups

Group	Animal	CBE treatment	N
1	A53T M83	Saline (i.p., once daily, 10 days)	6
2	A53T M83	CBE (1 mg/kg, i.p., once daily, 10 days)	9
3	A53T M83	CBE (5 mg/kg, i.p., once daily, 10 days)	9
4	A53T M83	CBE (25 mg/kg, i.p., once daily, 10 days)	9

B. Body weight over time

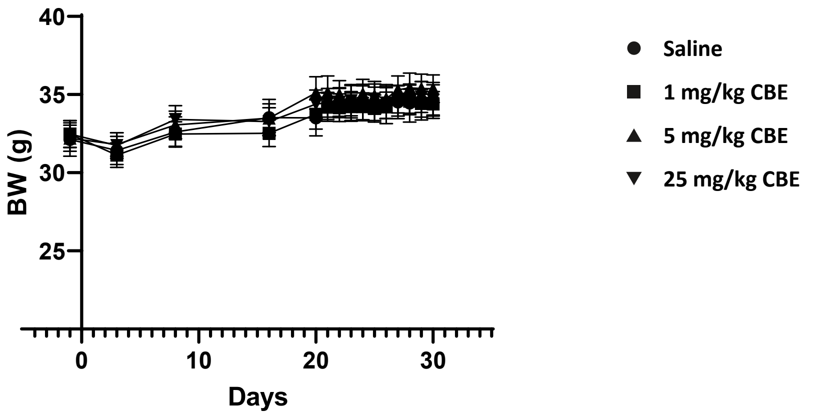

C. GCase activity in brain

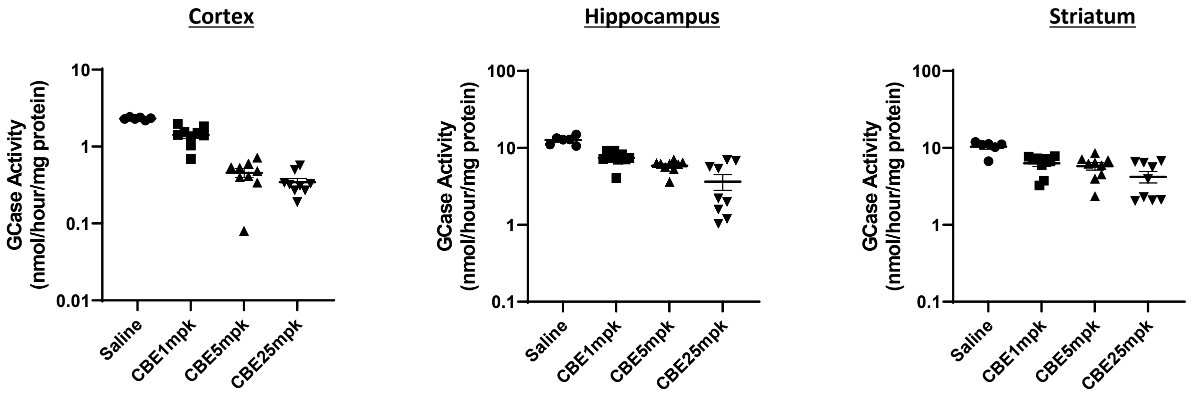

D. GlcSph accumulation

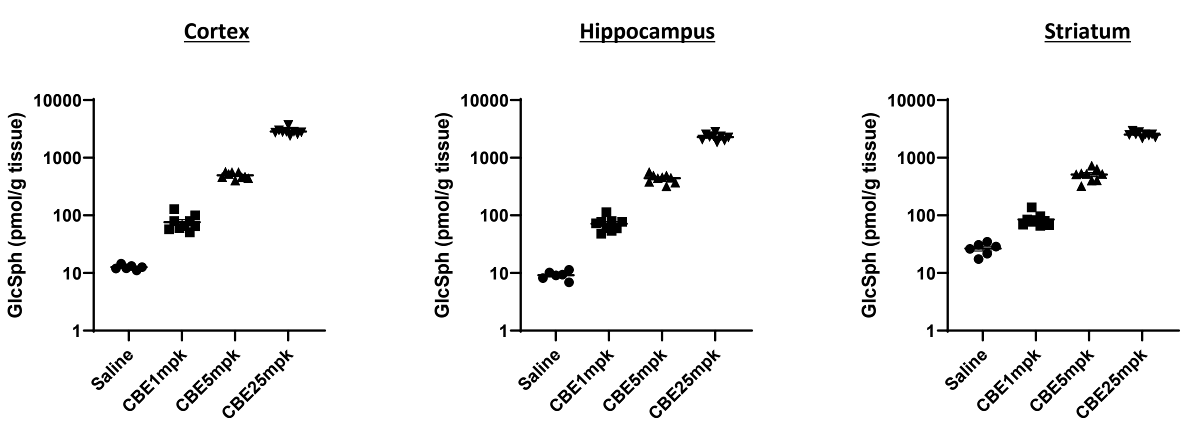

Supplement: S7 Fig — (A) Table for CBE dose finding. (B) Graph represents body weights per group over time. (C) GCase activity in cortex, hippocampus, and striatum was analyzed and the graph shows mean values per group, and (D) GlcSph levels were determined, see S7 and S8 Tables for mean values per group. The Y-axis is logarithmic scale. (PDF) [file pone.0321145.s007.pdf]
