# Supplementary material for: AAV delivery of GBA1 suppresses α-synuclein accumulation in Parkinson’s disease models and restores functions in Gaucher’s disease models
Source: PLoS One. 2025 May 7;20(5):e0321145. doi: 10.1371/journal.pone.0321145 (PMC12057913; doi:10.1371/journal.pone.0321145)

S8 Fig.

A. Body weight over time

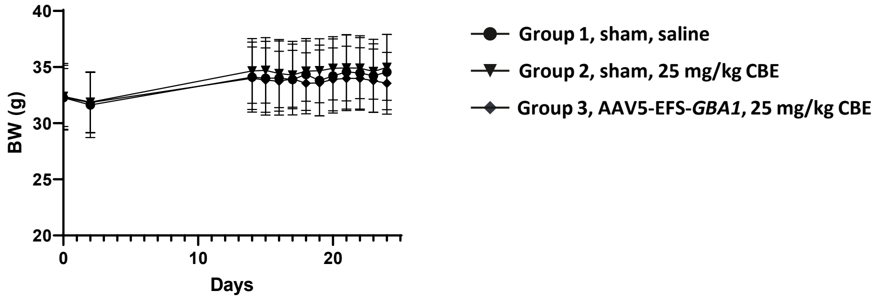

B. WES Image (Striatum)

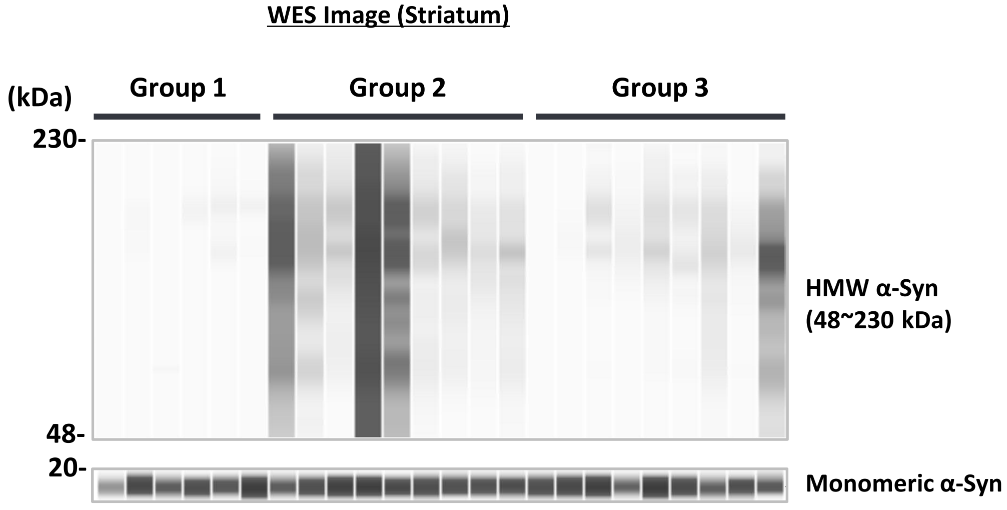

C. Monomeric α-Syn quantification (Striatum)

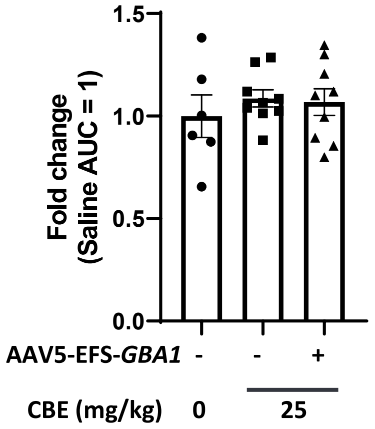

Supplement: S8 Fig — (A) Graph represents body weights per group over time. (B) Western (WES) images of monomeric (~19 kDa) and HMW α-synuclein (48–230 kDa) in Triton X-100-insoluble fraction of striatum from groups is shown. Each lane represents each mouse striatum sample data. (C) Graph represents AUC of monomeric α-synuclein normalized with the average of Group 1 by WES. Statistical analyses were performed by Dunnett analysis. *: < 0.05; **: < 0.01; ***: < 0.001 compared to group 2 (sham, CBE). (PDF) [file pone.0321145.s008.pdf]
