# Supplementary material for: AAV delivery of GBA1 suppresses α-synuclein accumulation in Parkinson’s disease models and restores functions in Gaucher’s disease models
Source: PLoS One. 2025 May 7;20(5):e0321145. doi: 10.1371/journal.pone.0321145 (PMC12057913; doi:10.1371/journal.pone.0321145)

S9 Fig.

A. WES image and HMW α-Syn quantification (Cortex)

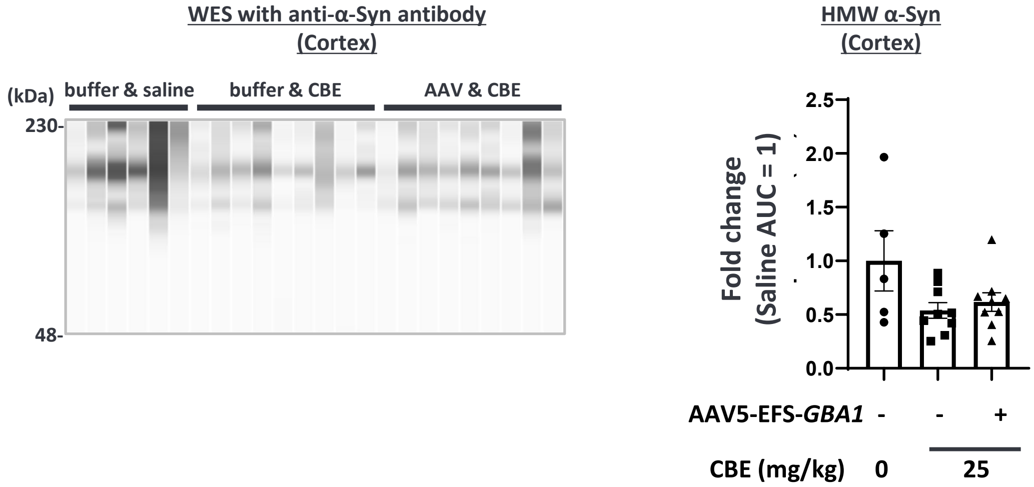

B. WES image and HMW α-Syn quantification (Hippocampus)

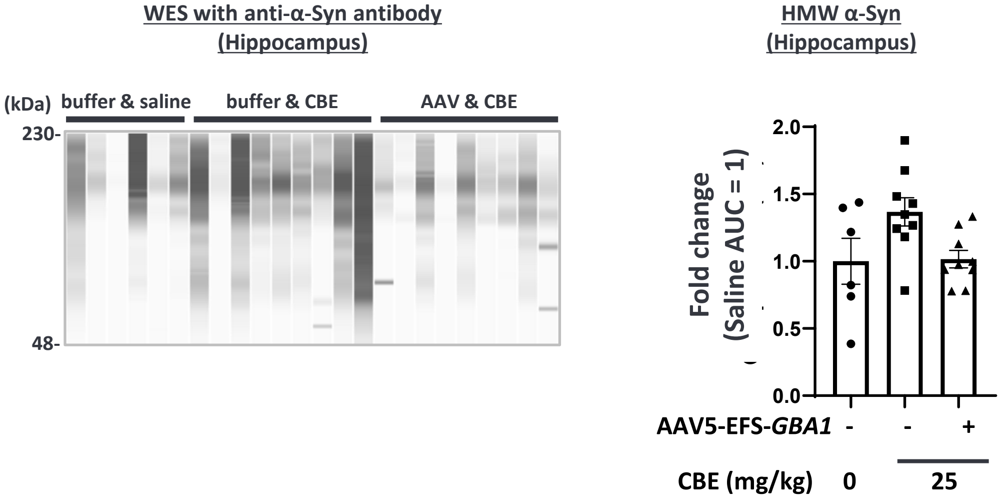

Supplement: S9 Fig — (A) Western (WES) image of HMW α-synuclein (48–230 kDa) in Triton X-100-insoluble fraction of cortex from groups is shown. Right graph represents AUC of HMW α-synuclein normalized with the average of Group 1 by WES. (B) WES image of hippocampus (left) is shown. Right graph represents AUC of HMW α-synuclein normalized with the average of Group 1 by WES. Statistical analyses were performed by Dunnett analysis. *: < 0.05; **: < 0.01; ***: < 0.001 compared to group 2 (sham, CBE). (PDF) [file pone.0321145.s009.pdf]
