# Supplementary material for: AAV delivery of GBA1 suppresses α-synuclein accumulation in Parkinson’s disease models and restores functions in Gaucher’s disease models
Source: PLoS One. 2025 May 7;20(5):e0321145. doi: 10.1371/journal.pone.0321145 (PMC12057913; doi:10.1371/journal.pone.0321145)
Supplement: S1 Table — lists the mean values ± S.E.M. for GCase activity per group and mean fold change for Fig 3B. (PDF) [file pone.0321145.s010.pdf]

S1 Table. Mean GCase Activity and Fold Change in Fig 3B.

**Mean GCase Activity ± SEM (nmol/h/mg protein) by AAV9-*GBA1***			**Mean Fold Increase in GCase activity relative to Group 2**
Group 1	Group 2	Group 3	Group 3
10.8 ± 0.5	5.9 ± 0.2	39.8 ± 12.8	6.7

**Mean GCase Activity ± SEM (nmol/h/mg protein) by AAV5-*GBA1***				**Mean Fold Increase in GCase activity relative to Group 5**	
Group 4	Group 5	Group 6	Group 7	Group 6	Group 7
15.1 ± 0.3	9.5 ± 0.2	18.5 ± 2.4	18.5 ± 3.2	1.9	1.9
