# Supplementary material for: AAV delivery of GBA1 suppresses α-synuclein accumulation in Parkinson’s disease models and restores functions in Gaucher’s disease models
Source: PLoS One. 2025 May 7;20(5):e0321145. doi: 10.1371/journal.pone.0321145 (PMC12057913; doi:10.1371/journal.pone.0321145)
Supplement: S2 Table — for the mean values ± S.E.M. for GlcSph levels per group and mean fold change for Fig 3C. (PDF) [file pone.0321145.s011.pdf]

S2 Table. Mean GlcSph Level and Fold Change in Fig 3C.

	Mean GlcSph level ± SEM (pmol/g tissue) by AAV9-*GBA1*			Mean Fold decrease in GlcSph accumulation relative to Group 2
	Group 1	Group 2	Group 3	Group 3
Brain	7.7 ± 0.7	459.1 ± 50.2	129.5 ± 57.4	3.5
Liver	105.1 ± 57.4	3210.5 ± 75.6	794.1 ± 559.8	4.0

	Mean GlcSph quantity ± SEM (pmol/g tissue) by AAV5-*GBA1*				Mean Fold decrease in GlcSph accumulation relative to Group 5	
	Group 4	Group 5	Group 6	Group 7	Group 6	Group 7
Brain	10.4 ± 1.0	458.1 ± 42.0	108.4 ± 22.8	130.5 ± 30.7	4.2	3.5
Liver	14.3 ± 8.6	3206.3 ± 236.1	2410.8 ± 295.7	3022.9 ± 189.2	1.3	1.1
