# Supplementary material for: AAV delivery of GBA1 suppresses α-synuclein accumulation in Parkinson’s disease models and restores functions in Gaucher’s disease models
Source: PLoS One. 2025 May 7;20(5):e0321145. doi: 10.1371/journal.pone.0321145 (PMC12057913; doi:10.1371/journal.pone.0321145)
Supplement: S3 Table — summarizes the mean value ± S.E.M. for GCase activity per group and mean fold change for Fig 4B. (PDF) [file pone.0321145.s012.pdf]

S3 Table. Mean GCase Activity and Fold Change in Fig 4B.

	Mean GCase Activity ± SEM (nmol/hour/mg protein)				Mean Fold Increase in GCase activity relative to Group 2	
	Group 1	Group 2	Group 3	Group 4	Group 3	Group 4
Cortex	1.55 ± 0.04	2.19 ± 0.07	6.80 ± 2.75	4.47 ± 0.85	3.1	2.0
Hippocampus	0.98 ± 0.03	1.23 ± 0.07	66.63 ± 24.03	78.99 ± 30.07	54.0	64.1
Striatum	0.77 ± 0.05	1.42 ± 0.08	2.76 ± 0.51	1.86 ± 0.20	1.9	1.3
