# Supplementary material for: AAV delivery of GBA1 suppresses α-synuclein accumulation in Parkinson’s disease models and restores functions in Gaucher’s disease models
Source: PLoS One. 2025 May 7;20(5):e0321145. doi: 10.1371/journal.pone.0321145 (PMC12057913; doi:10.1371/journal.pone.0321145)
Supplement: S4 Table — for the mean values ± S.E.M. for GlcSph levels per group and mean fold change for Fig 4C. (PDF) [file pone.0321145.s013.pdf]

S4 Table. Mean GlcSph Level and Fold Change in Fig 4C.

	Mean GlcSph quantity ± SEM (pmol/g tissue) or (pmol/mL in CSF)				Mean Fold decrease in GlcSph relative to Group 2	
	Group 1	Group 2	Group 3	Group 4	Group 3	Group 4
Cortex	1306.7 ± 27.9	7210.2 ± 251.0	3209.1 ± 342.4	3252.7 ± 429.6	2.2	2.2
Cerebellum	1317.1 ± 91.1	13011.9 ± 606.1	7863.5 ± 711.8	9675.0 ± 366.3	1.7	1.3
CSF	0.090 ± 0.020	0.695 ± 0.055	0.339 ± 0.067	0.427 ± 0.074	2.1	1.6
Liver	362.7 ± 15.3	5653.1 ± 327.3	5940.3 ± 318.6	6146.4 ± 224.2	1.0	0.9
