# Supplementary material for: AAV delivery of GBA1 suppresses α-synuclein accumulation in Parkinson’s disease models and restores functions in Gaucher’s disease models
Source: PLoS One. 2025 May 7;20(5):e0321145. doi: 10.1371/journal.pone.0321145 (PMC12057913; doi:10.1371/journal.pone.0321145)
Supplement: S5 Table — lists the mean values ± S.E.M. for GCase activity for Fig 6B. (PDF) [file pone.0321145.s014.pdf]

S5 Table. Mean GCase Activity and Fold Change in Fig 6B.

**Mean GCase Activity ± SEM (nmol/h/mg protein) by AAV9-*GBA1***			**Mean Fold Increase in GCase activity relative to Group 2**
Group 1	Group 2	Group 3	Group 3
6.7 ± 0.2	3.6 ± 0.4	10.5 ± 3.4	2.9
