# Supplementary material for: AAV delivery of GBA1 suppresses α-synuclein accumulation in Parkinson’s disease models and restores functions in Gaucher’s disease models
Source: PLoS One. 2025 May 7;20(5):e0321145. doi: 10.1371/journal.pone.0321145 (PMC12057913; doi:10.1371/journal.pone.0321145)
Supplement: S6 Table — lists the mean values ± S.E.M. for GlcSph levels per group and mean fold change for Fig 6C. (PDF) [file pone.0321145.s015.pdf]

S6 Table. Mean GlcSph Level and Fold Change in Fig 6C.

Mean GlcSph quantity ± SEM (pmol/g tissue)			Mean Fold decrease in GlcSph relative to Group 2
Group 1	Group 2	Group 3	Group 3
29.5 ± 0.7	2945.9 ± 257.6	1748.0 ± 257.0	1.7
