# Supplementary material for: AAV delivery of GBA1 suppresses α-synuclein accumulation in Parkinson’s disease models and restores functions in Gaucher’s disease models
Source: PLoS One. 2025 May 7;20(5):e0321145. doi: 10.1371/journal.pone.0321145 (PMC12057913; doi:10.1371/journal.pone.0321145)
Supplement: S7 Table — lists the mean values ± S.E.M. for GCase and fold change in S7C Fig. (PDF) [file pone.0321145.s016.pdf]

S7 Table. Mean GCase Activity and Fold Change in S7C Fig.

	Mean GCase Activity ± SEM (nmol/hour/mg protein)				Mean Fold decrease in GCase activity relative to Saline group		
	Saline	1 mg /kg CBE	5 mg /kg CBE	25 mg /kg CBE	1 mg /kg CBE	5 mg /kg CBE	25 mg /kg CBE
Cortex	2.32 ± 0.04	1.42 ± 0.13	0.46 ± 0.06	0.34 ± 0.04	1.6	5.1	6.7
Hippocampus	12.59 ± 0.65	7.40 ± 0.51	5.86 ± 0.33	3.65 ± 0.84	1.7	2.1	3.5
Striatum	10.38 ± 0.77	6.30 ± 0.56	5.78 ± 0.62	4.21 ± 0.71	1.6	1.8	2.5
