# Supplementary material for: AAV delivery of GBA1 suppresses α-synuclein accumulation in Parkinson’s disease models and restores functions in Gaucher’s disease models
Source: PLoS One. 2025 May 7;20(5):e0321145. doi: 10.1371/journal.pone.0321145 (PMC12057913; doi:10.1371/journal.pone.0321145)
Supplement: S8 Table — list the mean values ± S.E.M. for GlcSph and fold change in S7D Fig. (PDF) [file pone.0321145.s017.pdf]

S8 Table. Mean GlcSph Level and Fold Change in S7D Fig.

	Mean GlcSph quantity ± SEM (pmol/g tissue)				Mean Fold increase in GlcSph accumulation relative to Saline Group		
	Saline	1 mg /kg CBE	5 mg /kg CBE	25 mg /kg CBE	1 mg /kg CBE	5 mg /kg CBE	25 mg /kg CBE
Cortex	12.6 ± 0.5	76.2 ± 8.2	494.1 ± 20.2	2845.3 ± 122.8	6.0	39.2	225.9
Hippocampus	9.2 ± 0.6	71.4 ± 6.5	441.3 ± 24.6	2275.3 ± 96.6	7.8	48.1	248.1
Striatum	26.6 ± 2.6	84.8 ± 7.4	509.6 ± 40.9	2521.7 ± 80.6	3.2	19.1	94.7
